# Comprehension of medication-related information translated from Japanese to Southeast Asian Languages using google translate

**DOI:** 10.1186/s40780-026-00559-1

**Published:** 2026-03-19

**Authors:** Yumina Ako, Takafumi Sugawara, Sho Ishida, Kazuyuki Tanaka, Atsushi Watanabe

**Affiliations:** 1 Department of Pharmacy, Hiroshima City Hiroshima Citizens Hospital, 7-33 Motomachi, Naka-ku, Hiroshima, Hiroshima 730-8518 Japan; 2 Pharmacy Division, Hiroshima City Rehabilitation Hospital, Hiroshima, Hiroshima Japan

**Keywords:** The translation application, Non-Japanese patients, Southeast Asia, Language barriers, Google Translate

## Abstract

**Background:**

A workforce of foreign workers, mainly from Southeast Asia (SEA), is increasing because of an aging society and a declining birthrate. However, there are a few hospitals that can accept them. In this study, we evaluated whether SEA persons can understand medication-related information translated from Japanese into SEA languages using Google Translate.

**Methods:**

The study was a questionnaire survey of simulated cases. Information on simulated cases in Japanese was translated into each target language using Google Translate. We set the answer to the questionnaire based on the simulated cases. Participants’ backgrounds and answers to the questionnaire were aggregated for each target language and category.

**Results:**

The participants were 24 people from Thailand, Vietnam, Indonesia, and Burma. Comprehension outcomes differed across medications and information types. While understanding of main effects and side effects was generally adequate, comprehension of medication usage was limited, particularly for loxoprofen, with substantial variation across language groups.

**Conclusions:**

It’s challenging to communicate medical information accurately to the SEA patients when relying solely on Google Translate. Therefore, we may need to provide medication guidance orally to improve and enhance medication-related information, even if we cannot speak SEA languages.

## Background

In Japan, there are more than 2 million non-Japanese workers, who account for approximately 3.4% of the country’s workforce. The number of Japanese workers is expected to decrease due to an aging society and declining birthrate. Therefore, the Japanese cabinet believes that Japanese people need a workforce of foreign workers [1]. In Hiroshima Prefecture, there are many non-Japanese workers employed in the fisheries and manufacturing industries. The statistics on foreign workers in Hiroshima Prefecture indicate that the majority of the occupied population is from Vietnam [2, 3]. In this trend, the number of non-Japanese patients visiting hospitals is increasing nowadays, especially from Southeast Asia (SEA) [4, 5].

In Japan, only a limited number of hospitals accept foreign patients and offer translation services [6], such as translation devices (e.g., *POKETALK* (Sourcenext Corp., Tokyo, Japan)). In addition, some Japanese hospitals have introduced tools specifically designed to support communication with foreign patients [7]. In the global context, several studies have reported the use of translation applications for medical communication [8–10]. Some studies included a unique tool for communicating with patients who don’t speak the country’s language [11, 12]. However, to our knowledge, no studies have focused on SEA languages, and we were unable to identify any reports evaluating the accuracy of translating medication-related information from Japanese into SEA languages.

We hypothesized that pharmacists could use translation apps more easily than professional interpreters when providing medication-related information to SEA patients working in Japan. In Hiroshima, residents whose first languages are Thai, Vietnamese, Indonesian, and Burmese represent the largest groups among SEA people. Especially, these SEA people come to the Hiroshima Japanese Class to learn Japanese. The purpose of this study was to evaluate how Google Translate can be used in real-world clinical settings to support SEA patients. In this study, we evaluated people whose first languages were Thai, Vietnamese, Indonesian, and Burmese to determine whether translated medication-related information from Japanese to SEA languages is accurately understood.

## Methods

### Study setting

**Fig. 1:**
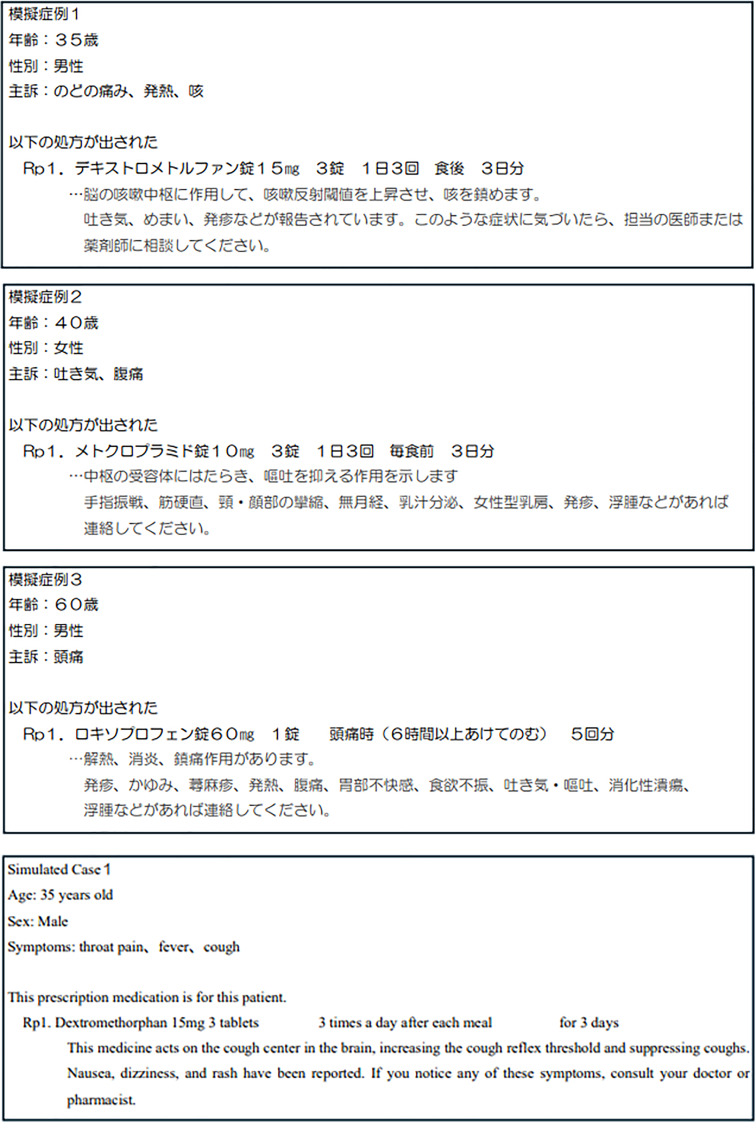

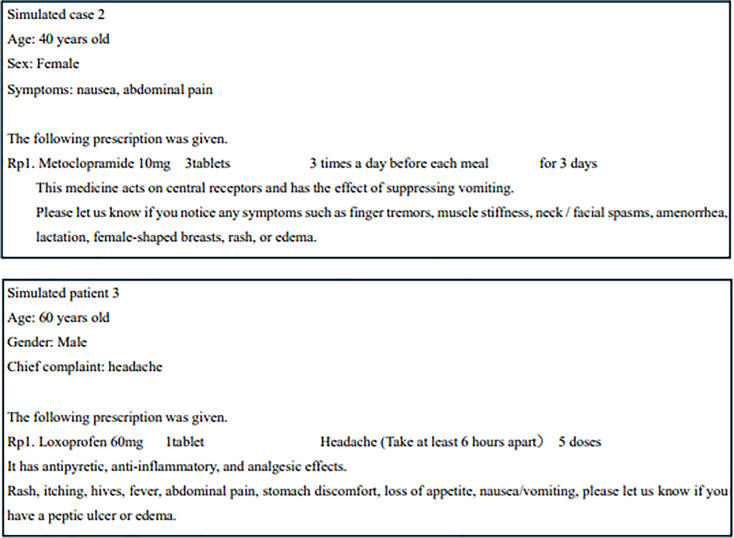
Original Japanese input and Google Translate output in simulated cases

The study was a questionnaire survey of simulated cases (Fig. 1). We use Google Translate in this study. Google Translate was used in this study between November 2024 and December 2024.

### Translation process

The simulated case texts were translated from Japanese into SEA languages solely using Google Translate. ChatGPT was not used to translate or modify the simulated case texts.

**Fig. 2:**
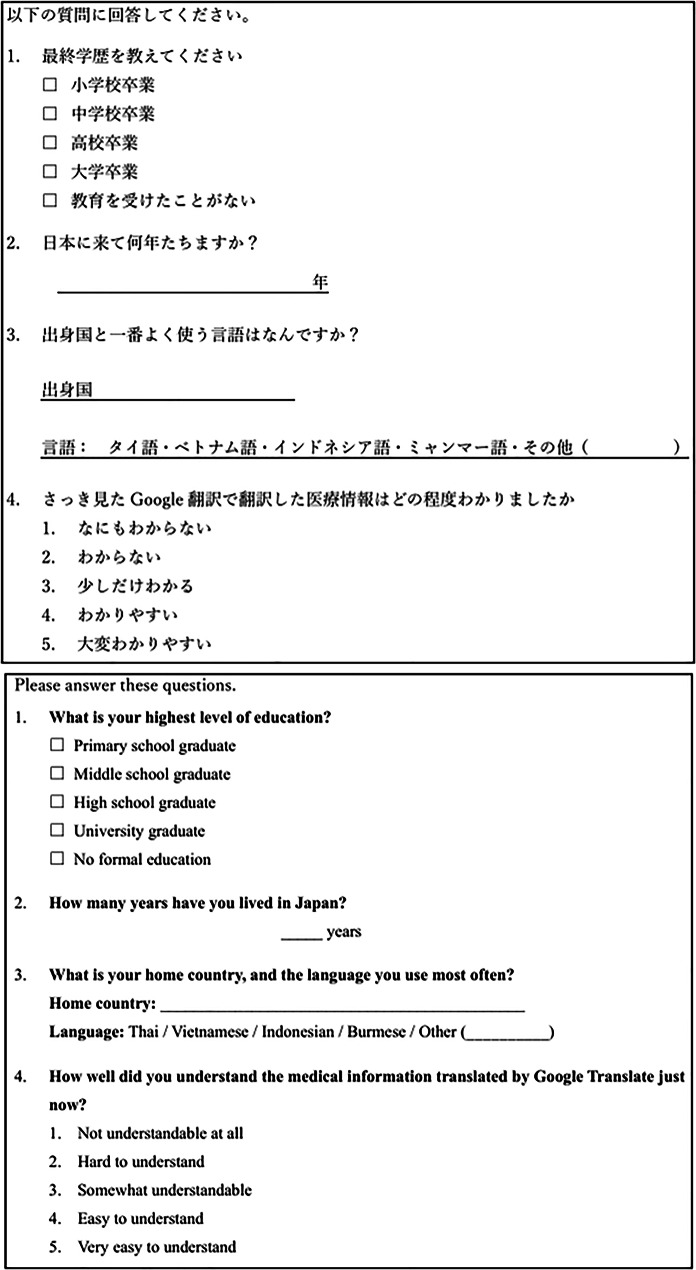
Original Japanese text and Google Translate output of the questionnaires assessing comprehension of translated medication-related information and participant characteristics

**Fig. 3:**
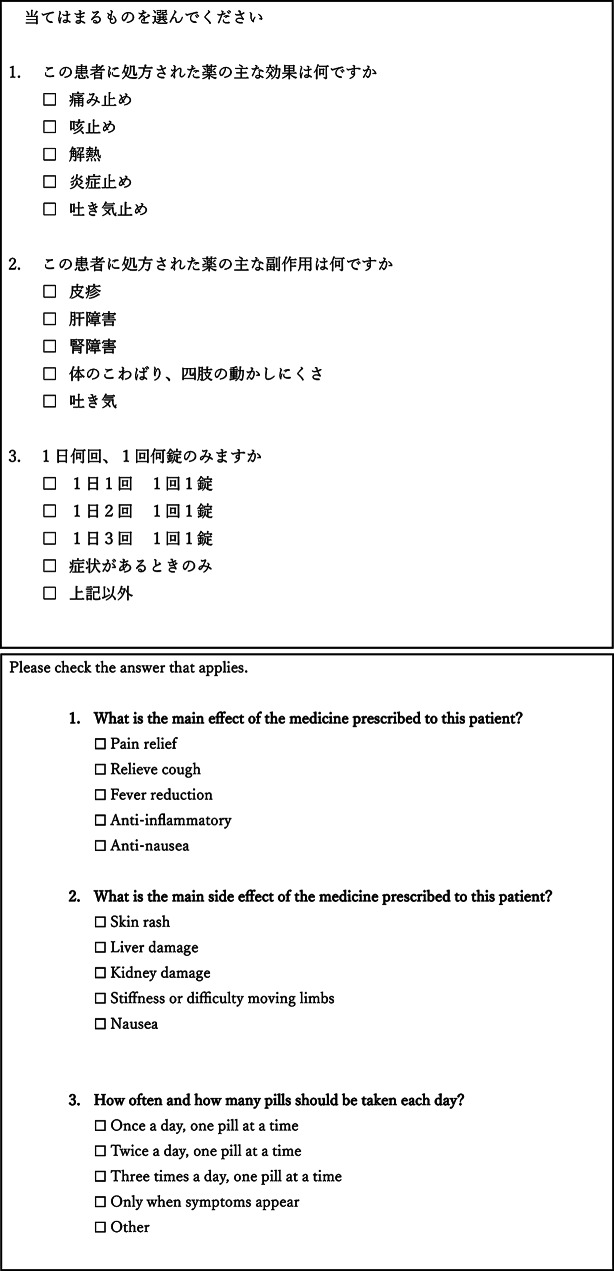
Original Japanese text and Google Translate output of questionnaire assessing comprehension in simulated cases

**Fig. 4:**
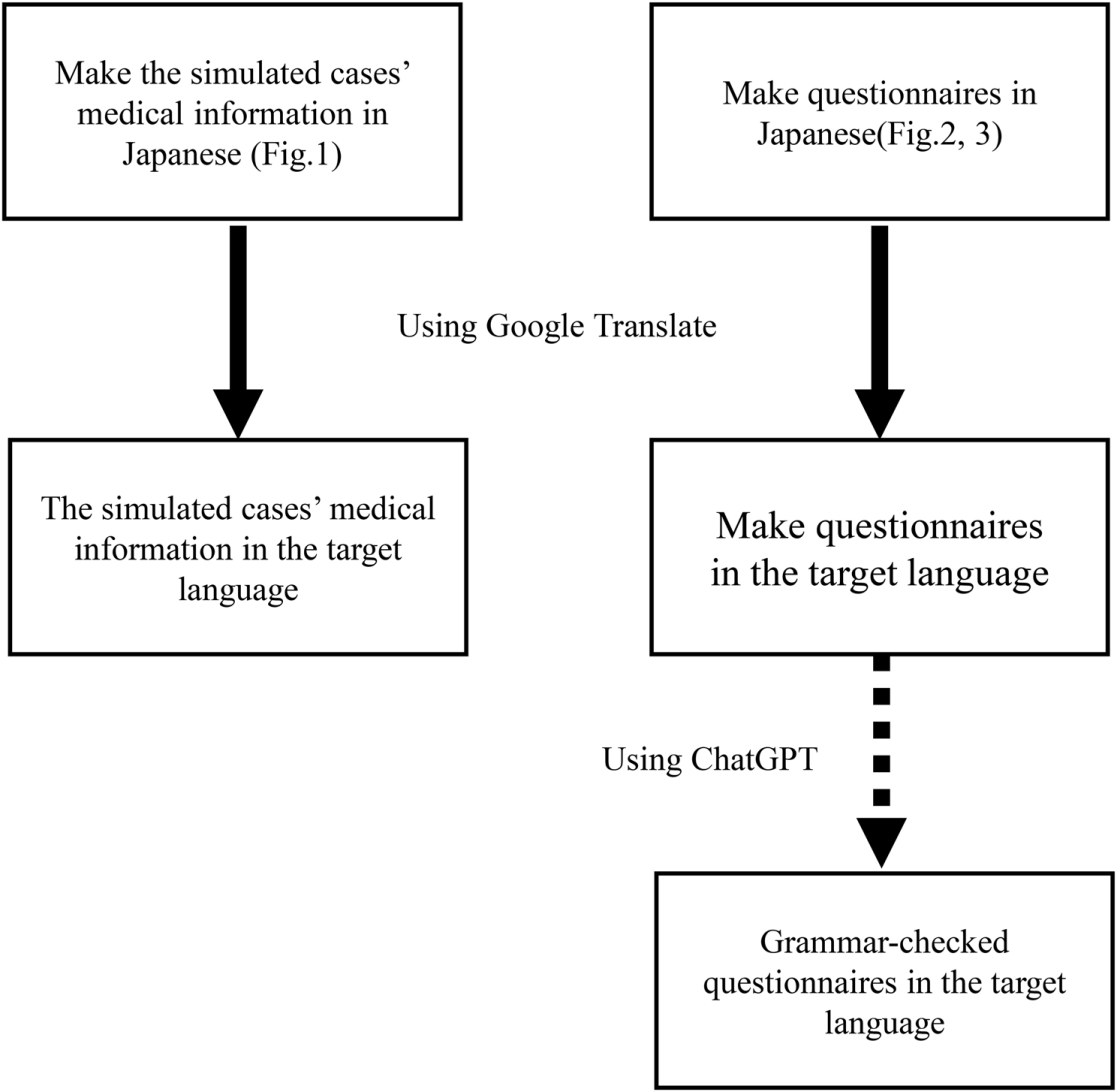
Algorithm of translation

To minimize grammatical errors in the questionnaire used to assess comprehension of the simulated case texts, we first prepared the questionnaire in Japanese (Figs. 2 and 3). The text was then translated using Google Translate, and the translated version was subsequently checked for grammatical errors using ChatGPT (GPT-4o) [13, 14] (Fig. 4).

### Sampling strategy

Because the number of SEA patients visiting our hospital was limited, we recruited simulated SEA patients for this study. Simulated SEA patients were individuals whose first language was a SEA language and who were able to participate in the evaluation of translated medication-related information. Simulated SEA patients’ languages are Thai, Vietnamese, Indonesian, and Burmese. These languages were selected because they represent the most commonly spoken SEA languages among residents attending community-based Japanese language classes in Hiroshima; therefore, the findings are intended to reflect this specific population rather than all SEA residents in Japan. All study participants provided written consent to participate. They speak target languages and were recruited through Japanese class coordinators, who provided contact information to teachers in the Hiroshima Japanese class. This study was conducted as an exploratory pilot study. Therefore, no formal sample size calculation was performed. Participants were recruited based on availability during the study period, and all eligible individuals who agreed to participate were included.

### Data collection

Before the questionnaire survey began, general project information was provided, including the study’s aim, the participation requirements, and the intended use of the results. After that, we conducted this survey for about 15 min.

Participants evaluated translated medication-related information using a five-level rating scale, where one was the poorest and five was the best. The questionnaires about simulated cases consisted of closed-ended questions and allowed multiple responses. The answers to the questionnaires were predetermined (Table 1). When participants were asked about our study, the researcher explained it to them in Yasashii Nihongo [15], a simplified form of Japanese for non-Japanese residents. In that case, no explanations or feedback were provided to participants during the comprehension assessment. The question-and-answer session was conducted only after the questionnaire was completed.

**Table 1:**
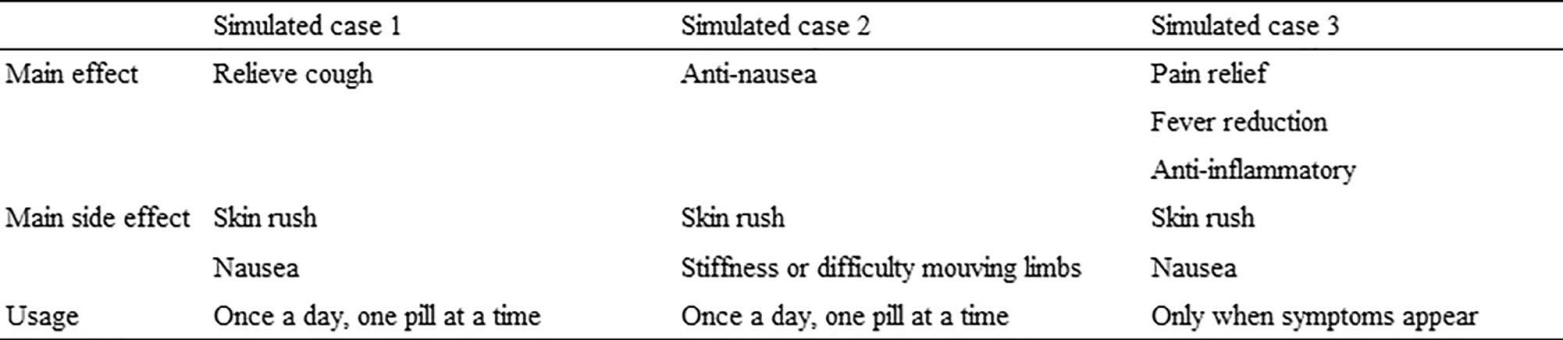
Setting answer of the simulated cases’ questionnaire

Correct answers were defined as selections consistent with the pharmacological effects described in the simulated case

### Data analysis and result reporting

The researcher collected data at the Hiroshima Japanese Class. The results of the translated answers were collected on paper. Participants’ backgrounds and answers to the questionnaire were collected for each target language and category. The duration of residence in Japan was collected in years. For participants who had lived in Japan for less than one year, the duration was recorded in months at the time of data collection. All values were subsequently converted to months for analysis.

Only participants who selected all researcher-defined correct options and no incorrect options were classified as having “adequate comprehension.” Any selection including one or more incorrect options was considered insufficient understanding from a medication-safety perspective. Participants may find it difficult to answer questions about hypothetical medical situations that they have not personally experienced. Because the simulated cases described multiple main effects, participants who selected no incorrect answers were considered to have acceptable comprehension.

We reported the results using descriptive statistics due to a small sample size.

### Ethical considerations

This study was approved by the Hiroshima City Hiroshima Citizens Hospital Ethics Review Committee (Application No. 2024 − 147). It was conducted in accordance with the *Ethical Guidelines for Medical and Biological Research Involving Human Subjects* and the principles of the *Declaration of Helsinki*. Confidentiality, neutrality, anonymity, accountability, and academic honesty were maintained throughout the study.

### Consent to participate

Informed consent was obtained from all participants prior to their participation in the study. Participants were informed that participation was voluntary and that refusal to participate would not result in any disadvantage.

## Results

### Characteristics of study participants

In total, 24 participants were included. Participants originated from Thailand, Vietnam, Indonesia, and Burma, and all were native speakers of their first languages. Among the participants, Vietnamese and Indonesian individuals accounted for the largest proportion. The length of residence in Japan ranged from 0 to 180 months, with most participants residing for less than one year. Educational backgrounds varied, and 5 of 7 Vietnamese participants had no formal education (Table 2).

**Table 2:**
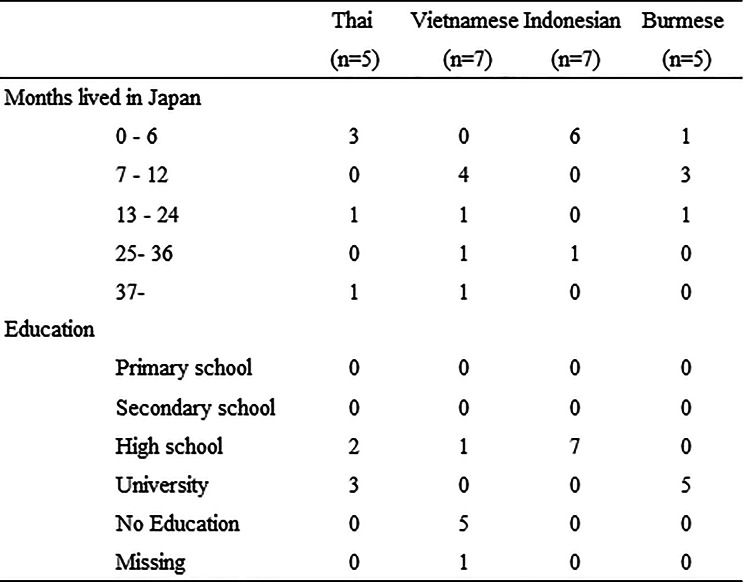
Characteristics of study participants

### Five-level rating scale of translated medication-related information

The median values were identical between the Vietnamese and Indonesian groups; however, the Indonesian group showed higher values compared to the Vietnamese group. The median value was lower in the Thai and Burmese groups than in the other groups. Although the median values were identical between the Thai and Burmese groups, the Burmese group demonstrated a wider range, including lower values (Table 3).

**Table 3:**
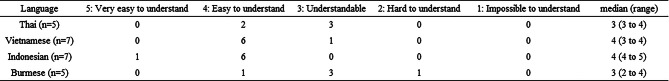
Self-assessment of understanding for translated medication-related information using five-level scale

### The result of understanding about simulated cases

Comprehension outcomes differed across the three medications. The key results are summarized below, with detailed results for each medication shown in Table 4.

#### Comprehension of the main effects

Among Thai participants, 3 of 5 and 5 of 5 selected all researcher-defined correct options of dextromethorphan and metoclopramide, respectively. Similarly, among Vietnamese participants, all researcher-defined correct options were frequently selected for these two medications. Among Burmese participants, 1 of 5 did not respond; however, among the 4 who responded, several selected the researcher-defined correct options for both medications. In contrast, 3 of 7 and 4 of 7 Indonesian participants selected all researcher-defined correct options for the main effects for dextromethorphan and metoclopramide.

For Loxoprofen, participants across all language groups frequently selected no incorrect options; however, no participants selected all researcher-defined correct options.

#### Comprehension of the side effects

Across all language groups and simulated cases, most participants selected no incorrect options. However, only a limited number of participants selected all researcher-defined correct options.

#### Comprehension of the usage

In simulated cases of dextromethorphan and metoclopramide, many participants across all language groups selected no incorrect options and all researcher-defined correct options.

In contrast, for Loxoprofen, 2 of 5 Thai and 6 of 7 Vietnamese participants selected “The other” option. Although 5 of 7 Indonesian participants selected “only when symptoms appear,” which was the researcher-defined correct option, comprehension of usage remained limited overall. Among Burmese participants, one participants selected the researcher-defined correct option.

**Table 4:**
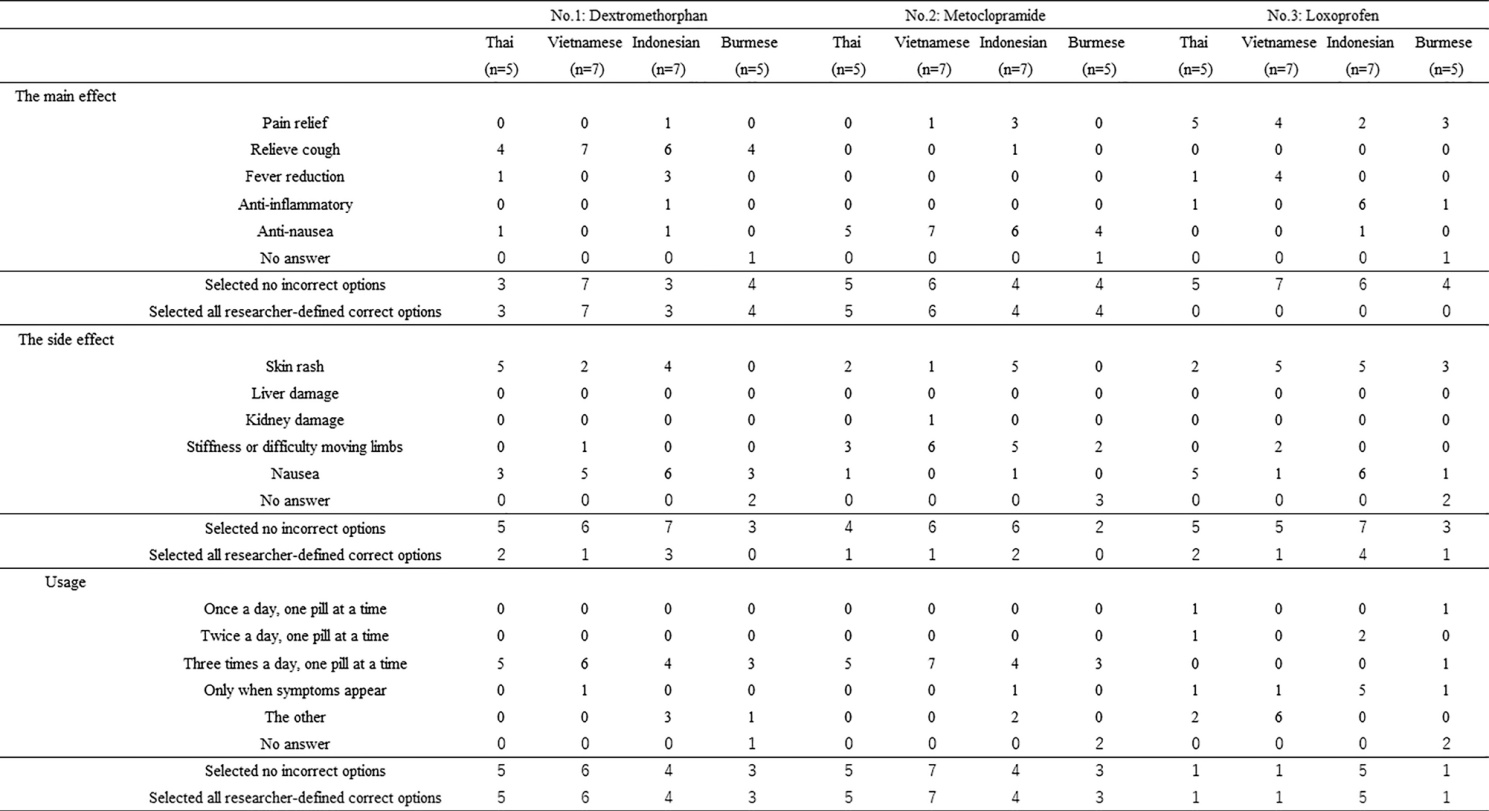
Result of simulated case

1. Because multiple responses were allowed, the total number of selected options may exceed the number of participants

2. Among participants who selected no incorrect options, the number of those who selected all researcher-defined correct options is also shown

## Discussion

The results did not demonstrate the expected benefit of using Google Translate when Japanese pharmacists present medication-related information to SEA patients from a medication safety perspective. However, these findings provided important insights into the limitations of using Google Translate for SEA patients.

In this study, the Indonesian and Vietnamese comprehensive scores were higher than those of the Thai and Burmese (Table 3). This is likely to be caused by the system of Google Translate. Google Translate is an easily accessible, free-to-use, and available in many languages worldwide. Obviously, this application includes SEA languages: Thai, Indonesian, Vietnamese, Lao, Burmese, Filipino, and Qumail. Google Translate is used by the Multilingual Neural Machine Translation (NMT) model [16]. The accuracy of NMT model translation apps increases with the amount of high-quality language data available on the internet. There are only two languages spoken by Indonesians (252,400,000) and Vietnamese (97,000,000) in the top 20 most spoken languages [17]. That means the accuracy of translation from Japanese to Indonesian and Vietnamese is higher than that to Thai and Burmese, which are not widely used on the internet.

Despite this, a discrepancy was observed between the five-level rating scale scores and actual comprehension of the simulated cases. In particular, Indonesians tended to select incorrect options more frequently than the other language groups in the main effect and usage sections of dextromethorphan and metoclopramide, even though they reported that the translated medical information was easy to understand (Table 3). This finding was expected to be related to participants’ educational background and length of residence in Japan; however, this study was not designed to formally evaluate these relationships. We had expected that participants with longer periods of residence and those with university education would show a stronger tendency to select only the correct answer. However, Vietnamese respondents exhibited a high rate of selecting only the correct answer despite lacking formal education. Therefore, a common factor among these groups, being older and having more social experience, was assumed to be a potential reason for their ability to select only correct answers. However, since respondents’ ages were not recorded in this survey, these remain possibilities only.

This study has several limitations.

First, simulated cases were used instead of real-world patients. The use of simulated cases was necessary due to the limited number of available cases and the difficulty of recruiting participants from clinical departments. The primary aim of this study was to explore the feasibility of evaluating translated medication-related information among speakers of Thai, Vietnamese, Indonesian, and Burmese, who are numerous residents in Hiroshima. Although simulated cases were used, the study may still have relevance to real-world clinical settings.

Second, as this was a pilot study, the sample size was not predetermined. In Japan, the number of SEA patients has been increasing year by year, requiring Japanese pharmacists to devote more time and resources to explaining medication-related information. Although exploratory, this study may provide preliminary insights that could support pharmacists in explaining medication-related information to SEA patients in real-world clinical settings. Future studies should recruit real-world patients and enroll a large number of participants to further validate these findings.

Third, a limitation of this study is that participants’ Japanese-language proficiency was not formally assessed. At the study design stage, we aimed to evaluate patients’ comprehension of translated medication-related information in real-world clinical settings. So we didn’t include language proficiency assessments in this study setting. As a result, age, detailed life experience, and Japanese-language proficiency were not collected. Several factors, including differences in educational background and unmeasured factors such as age, may have influenced the observed variability in comprehension. However, the potential influence of these factors could not be fully evaluated in this study. Future studies will collect detailed background information and clarify the relationship between patients’ backgrounds and their comprehension of translated medication-related information.

Fourth, we did not verify whether the expressions used in the simulated cases were culturally equivalent across countries or specific to Japanese medical contexts. In this study, dosage information was presented using Japanese prescription conventions (daily dosage). This may have affected participants’ responses to questions about medication use. In addition, this study was intentionally designed to avoid the involvement of native speakers or language assistants in order to reflect real-world. All translations were generated exclusively using Google Translate. Consequently, we did not assess the linguistic accuracy or appropriateness of the translated phrases as judged by native speakers.

Fifth, in the question about loxoprofen use, the response options may have influenced participants’ choices. In particular, the wording and structure of the options may partly explain the observed differences between Indonesian and Thai, and between Indonesian and Vietnamese. Therefore, the interpretation of the apparent reversal in comprehension across language groups should be made with caution.

Additionally, a language barrier between researchers and participants may have influenced responses, although the extent of this effect could not be evaluated. Even though explanations were provided in Yasashii Nihongo and no feedback was given during the comprehension assessment, this barrier limited our ability to probe participants’ reasoning processes or to clarify the reasons for misunderstandings. Nevertheless, because participants received no additional explanation or interpretive support during the assessment, the responses may reflect comprehension of the translated text itself.

Therefore, it is dangerous to rely solely on Google Translate when presenting medication-related information from a medication safety perspective. But we consider a real-world situation in which we can only use Google Translate. In actuality, Japanese community pharmacies often use English or Yasashii Nihongo to explain to foreign patients how to use their medications [18]. Wang noted that language assistance, including direct conversations, AI translators, and interpreters, can increase understanding about non-Japanese people’s illnesses [19]. Kido stated that Yasashii Nihongo is one of the tools for non-Japanese people to better understand medical information [20]. Based on these findings, to improve comprehension of medical information among SEA patients, practical examples include Yasashii Nihongo and Google Translate that any pharmacist can use easily and at no cost. In the future, we plan to investigate whether combining drug information generated with Google Translate with oral guidance in Yasashii Nihongo will enhance real-world SEA patients’ understanding of medications.

## Conclusions

It’s challenging to accurately communicate medication-related information to SEA patients when we rely solely on Google Translate. Previous studies have shown that the use of Yasashii Nihongo can improve patients’ understanding of medication-related information, even when pharmacists and SEA patients do not use the same language [20]. Our findings suggest that combining oral explanation with Yasashii Nihongo and translation applications may further support communication with SEA language speakers.

## Data Availability

Not applicable.

## Abbreviations

SEA: Southeast Asia
NMT: The Multilingual Neural Machine Translation
